# Gene-Drug Interaction in Stroke

**DOI:** 10.4061/2011/212485

**Published:** 2011-11-10

**Authors:** Serena Amici, Maurizio Paciaroni, Giancarlo Agnelli, Valeria Caso

**Affiliations:** Stroke Unit, Division of Cardiovascular Medicine, University of Perugia, Santa Maria della Misericordia Hospital, Sant'Andrea delle Fratte, 06126 Perugia, Italy

## Abstract

Stroke is the third cause of mortality and one of most frequent causes of long-term neurological disability, as well as a complex disease that results from the interaction of environmental and genetic factors. The focus on genetics has produced a large number of studies with the objective of revealing the genetic basis of cerebrovascular diseases. Furthermore, pharmacogenetic research has investigated the relation between genetic variability and drug effectiveness/toxicity. This review will examine the implications of pharmacogenetics of stroke; data on antihypertensives, statins, antiplatelets, anticoagulants, and recombinant tissue plasminogen activator will be illustrated. Several polymorphisms have been studied and some have been associated with positive drug-gene interaction on stroke, but the superiority of the genotype-guided approach over the clinical approach has not been proved yet; for this reason, it is not routinely recommended.

## 1. Introduction

Stroke is the third cause of mortality and one of most frequent causes of long-term neurological disability. Well-established risk factors for stroke include increasing age, hypertension, diabetes mellitus, cigarette smoking, obesity, heart disease, atrial fibrillation and sedentary [1, 2]. However, a significant number of patients experience stroke in the absence of any risk factors; a hypothesis is that many risk factors have not been recognized yet, including genetic risk factors. The role of genetics has been evidenced through studies on twins and family history. Twin studies have shown that monozygotic twins are 1.6 more likely to be concordant for stroke than dizygotic twins [3]. Family history of stroke is a well-defined risk factor (OR 1.76 95% CI 1.7–1.9) [3]. 

Given these data, genetic studies have increasingly been performed with the objective of revealing the genetic basis of cerebrovascular diseases. Genetic studies have been proposed to (1) reveal the pathogenetic basis of stroke, which might become a therapeutic target for new drugs, (2) optimize risk assessment, (3) identify populations requiring more aggressive therapeutic strategies, and (4) choose the optimal drug therapy by assessing the risk/benefit ratio based on genetic characteristics [4]. The latter application has been extensively studied in pharmacogenetic studies [5–7]. Recently, genetic studies have moved to “pharmacogenomic” that involve a genome-wide association approach which scans the entire genome looking through thousands of genetic variants; these hypothesis-free studies have the aim of discovering novel genes associated with a specific disease. This review has the aim of reporting on the latest developments regarding pharmacogenetics and pharmacogenomics of stroke, focusing on the most commonly used drugs in the acute phase, for primary and secondary prevention.

## 2. Methods

This review was planned using key words such as “pharmacogenetics” or “pharmacogenomics” and “stroke” to search literature. These words were combined with “antihypertensive agents,” “statins,” “hydroxymethylglutaryl-CoA Reductase Inhibitors,” “tissue plasminogen activator,” “anticoagulants,” “vitamin K antagonist,” “antiplatelets,” “cyclooxygenase Inhibitors,” “aspirin,” “clopidogrel,” and “acetil salicylic acid/dipyridamole.”

The following electronic databases were searched: MEDLINE (1995-June 11 2011) and EMBASE (1995-June 11 2011). One of the researchers (SA) read all the abstracts and selected all articles that included either “stroke” as outcome in primary prevention studies or as the target population in acute stroke treatment or secondary prevention studies. If any doubt was raised on an article's relevance, a second opinion was formulated by VC.

## 3. Results

In this section, pharmacogenetic studies involving drugs currently used for ischemic stroke (prevention or acute phase therapy) are reviewed.

### 3.1. Antihypertensive Agents

Hypertension is the most common stroke risk factor [41]. *β*1 and *β*2 adrenergic receptor (AR) plays a major role in cardiac disease; their codifying genes have been associated with response to antihypertensive drugs. **β*1-AR* gene interacted with beta-blocker (BB) therapy. Stroke risk has been shown to be higher in rs#2429511 carriers treated with BB (OR: 1.24, 95% CI: 1.03–1.50). On the contrary, BB therapy did not interact with **β*2-AR* gene variants on the risks of ischemic stroke (Table 1) [14]. A large randomised trial on treated hypertensive patients, enrolled to add either verapamil SR or trandolapril (International Verapamil SR-Trandolapril Study, INVEST study), focused on the genetic component of hypertension (INVEST-GENES) (Table 1) [8, 9, 17, 18, 20]. One of the papers derived from this study examined the polymorphism of **α*-adducin* (*ADD1*) *Gly460Trp* and race. The authors chose this polymorphism because *α*-adducin, a cytoskeleton protein related with sodium sensitivity and diuretics efficacy, has been linked to essential hypertension [42]. The results did not evidence any diuretic-genotype interaction [20]. On the contrary, a population-based case control study on the same polymorphism found that diuretics protected *ADD1 460 Trp* carriers from combined nonfatal MI/nonfatal stroke outcome. Other antihypertensive agents (e.g., beta blockers, ACE inhibitors, and calcium-channel blocker) did not show the same effect [19].

The randomised INVEST-GENES study also investigated the relation between subunit *β*1 of the gene that encodes for a conductance calcium and voltage-dependent potassium channel (*KCNMB1*) genotype and response to calcium antagonists. The results showed that carriers of the *Leu 110* polymorphism have a reduced risk of combined death, MI, and stroke when assuming verapamil SR to treat hypertension [9]. In addition, the same research group focused on G-protein-coupled receptor kinases (GRKs), receptors involved in beta-adrenergic signalling. *GRK2* SNPs (*rs1894111 G* > *A*) and *GRK5 Gln41Leu* polymorphism were investigated in patients treated with atenolol or hydrochlorothiazide. The authors concluded that *GRK 41Leu* variant did not interact with any of the studied treatment regarding a combined cardiovascular outcome including death, MI, and stroke [8]. Finally, Pacawnosky investigated for an association between nitric oxide synthase (*NOS 3*) polymorphism [18], beta-adrenergic receptor gene (*ADRB1* and *ADRB2*) [17], and response to different antihypertensive agents. The first study focused on two *NOS 3* polymorphisms since nitric oxide regulates vascular tone and is associated with many cardiac diseases [43]; no outcome or drug interaction was associated with genotype [18]. Also the second study did not evidence any genotype-drug interaction on stroke [17].

A population-based prospective cohort study focused on the renin-angiotensin system which is affected by ACE-inhibitors and BB (Table 1) [15, 16]. Neither of the studies observed any interaction between drug use and genotype when stroke was considered as outcome [15, 16].

The genetics of hypertension-associated treatment (GenHAT) study investigated the ACE insertion/deletion (*ACE I/D*) polymorphism in a large population of hypertensive patients with one or more cardiovascular risk factors. This randomised study did not report any association between treatment, genotype, and primary or secondary outcomes [11].

The same result was replicated in a more articulated investigation on the *ACE* gene and 12 other polymorphisms (*ADD1 Gly460Trp, *β*1AR Gly389Arg, *β*2AR Arg16Gly, *β*2AR Gln27Glu, *β*3AR Trp64Arg, AGT Met235Thr, Aldosterone synthase promoter C-344T, Type 1 angiotensinogen receptor A1166C, bradykinin 2 receptor I/D,* *CYP*2*C*9∗2* versus* *CYP*2*C*9∗1, *CYP*2*C*9∗3* versus* *CYP*2*C*9∗1*, G protein *β*3-subunit C825T*) [10]. This study was the product of the randomised LIFE (Losartan Intervention for Endpoint reduction in Hypertension) study trial, which included patients with hypertension and left ventricular hypertrophy treated with losartan versus atenolol. The authors did not evidence any genetic-drug interaction on different outcomes such as blood pressure and heart rate control, composite adverse cardiovascular outcome, cardiovascular death, MI, and stroke; in fact, they concluded that the clinical superiority of losartan in 25% stroke reduction compared to atenolol was not explained by these susceptibility genes [10]. 

A role in modulating antihypertensive agents has been suggested for the gene which codes for the precursor of atrial natriuretic polypeptide (*NPPA* gene). The polymorphism of this gene was studied by the GenHAT study [12]. The objective was to demonstrate that minor *NPPA* alleles in the *T2238C* or *G664A* variants had lower rates of primary outcome events compared with common allele homozygotes, if treated with diuretics. Subjects randomly receiving amlodipine, chlorthalidone, lisinopril, or doxazosin were included in a genetic for treatment interaction analysis. Carriers of the minor *C* allele had more favourable stroke outcome when taking diuretics, whereas *TT* allele carriers had better stroke outcome when receiving a calcium channel blocker [12]. GenHAT [13] also showed that stroke risk was higher on lisinopril versus amlodipine in common *GG* homozygotes of fibrinogen beta (*FGB*) gene, which codes for a polypeptide of the coagulation factor fibrinogen. On the contrary, variant *A* allele carriers on lisinopril had slightly lower stroke risk. Finally, a pharmacogenetic study on perindopril failed to demonstrate a role for ACE I/D polymorphism on stroke [21].

### 3.2. Statins

The most currently used drugs for hypercholesterolemia are statins; although very effective, they induce a significant rate of adverse events such as myopathies and abnormal transaminase levels. Recent pharmacogenetics data has contributed to better understanding statin pharmacokinetics and pharmacodynamic variability [44]. Pharmacogenetic and dynamic properties have been extensively studied, but only few studies included stroke as outcome (Table 2) [22–25].

A population-based cohort study focused on apolipoprotein E, a protein involved in lipid clearance rate and conversion together with the production of triglycerides and very low-density lipoprotein. The Apo E gene encodes for three alleles: E2, E3, and E4 [45]. The results did not show gene-statin interaction with stroke; stroke risk was reduced independently of Apo E genotype in statin users [24]. The same author examined the effect of *ACE I/D* polymorphism on stroke using the Gen-HAT data. None of the outcomes evidenced significant ACE I/D-pravastatin interaction [25]. The randomised heart protection study focused on Kinesin family member 6 (*KIF*) gene, whose variant has been associated with reduced coronary events [46]. The authors did not find any significant interaction between the studied polymorphism *KIF Trp719Arg* and simvastatin use for any of the outcomes, including stroke [23]. The only study that yielded positive results was a case-control study that involved patient with MI and stroke. The authors focused on six genes that have been associated to statin treatment response: ATP-binding cassette subfamily B (*ABCB1*) gene that encodes for a drug transporter involved in statins metabolism; *CETP*, human hepatic lipase gene (*LIPC*) and low density lipoprotein receptor (*LDLR*), genes related to lipid metabolism; *HMGCR*, the target protein of statins; *NOS3*, a key gene implicated in maintaining the endothelium, which in turn mediates several effects of statins [22]. The authors found 5 polymorphisms (one in CETP and 1 in LIPC) that had significant interactions with statins on stroke outcome [22], the highest significance level was found in the CETP SNPs (rs5883), which was associated with stroke risk in simvastatin users. No gene level interactions were found for stroke [22].

### 3.3. Tissue Plasminogen Activator

Recombinant tissue plasminogen activator (rTPA) is the only licensed drug to treat ischemic stroke in the acute phase (within 3–4.5 hours from onset). This drug is administered to treat ischemic stroke and restore blood flow to the brain [47, 48]. The clinical benefit of rTPA is counterbalanced by a higher risk of hemorrhagic complications; 2–10% of patients develops symptomatic hemorrhagic transformations and 40% asymptomatic hemorrhagic events [49–51]. The functional role of rt-PA is to convert plasminogen into plasmin, which has fibrinolytic activity. The higher activity of the enzyme produces hyperfibrinolysis and consequently bleeding, whereas lower activity causes hypofibrinolysis and, as a consequence, thrombosis or embolism [52]. Genetic association studies have sought to investigate genetic profiles correlated with clinical and pathophysiological rt-PA response (Table 3). Broderick et al. [26, 53] examined the role of the *ApoE* phenotypes and reported that rt-PA efficacy was greater in acute stroke patients with an *ApoE E2* phenotype (OR: 6.4; 95%, CI: 2.7–15.3), whereas the outcome of placebo-treated patients with or without *Apo E E2* did not differ [26]. Conversely, a Spanish group did not report on any association with *Apo E* genotype and hemorrhagic risk and recanalisation rate after thrombolytic treatment [27]. The same group explored the hypothesis that matrix metalloproteinase-9 gene (*MMP-9*), which codes for proteins associated with blood-brain barrier disruption, was associated with hemorrhagic transformation in rTPA-treated patients. However, the authors did not find any association between a *MMP-9 C-1562T* common polymorphism and hemorrhagic risk [32]. On the other hand, the authors reported that thrombolytic intervention yielded middle cerebral artery (MCA) recanalisation associated with DD homozygosis of ACE I/D polymorphism; this has been linked to procoagulant factors including PAI-1, fibrinogen's levels as well as Factors VII and X activities [29]. Another study of the same group has identified *V34L factor XII* polymorphism as a predictor of outcome with rTPA treatment; good outcome was associated with *VV* genotype and low fibrinogen levels, while a higher risk of inefficacy of thrombolytic therapy and mortality was found with *L34* genotype and high fibrinogen levels [31]. In addition, Fernandez-Cadenas and colleagues studied the influence of two genes coding for fibrinolysis inhibitors, thrombin-activatable fibrinolysis inhibitor (*TAFI*), and plasminogen activator inhibitor-1 (*PAI-1*) genes. They demonstrated that *TAFI Thr325Ile* polymorphism predicted the absence of recanalisation with t-PA infusion. On the contrary, *PAI-1 4 G/5 G* polymorphism did not influence recanalisation rate. However, the combination of these two polymorphisms doubled the risk of negative response to therapy [30]. A recent study using a candidate gene approach has explored the association of 263 SNPs and recanalisation rate in TPA-treated patients; cluster of differentiation 40 (CD40) 1C > T and matrix Gla protein (MGP)-7*A* > *G* polymorphism were both associated with reocclusion although only the latter was associated with neurological worsening at 24 h [28]. This may be due to the role of CD40 in thrombosis and inflammation [54], while MGP gene might have a protective role in atherosclerosis [55]. To date, GWAs has not been performed on human subjects.

### 3.4. Anticoagulants

Anticoagulation is first-line treatment for cardioembolic stroke. Although these drugs are effective in almost 60% of cases, the hemorrhagic risk is double and even higher in the first period of therapy [1]. Recent acquisition on pharmacogenetics of warfarin has been suggested to be able to predict the optimal initial dosage of warfarin using a genotype-guided approach (Table 4). This approach promises to adequately prevent stroke and to minimize hemorrhagic risk. Several candidate gene studies have mainly focused on cytochrome P450 (CYP) and vitamin K epoxide reductase complex subunit 1 (VKORC1) [56, 57]. Cytocrome P 450 metabolises in the liver S-warfarin by CYP2C9 and R-warfarin by the CYP1A1, CYP1A2, and CYP3A4; these enzymes affect warfarin kinetics, and several SNPs of CYP450 have been correlated with its sensitivity [58]. The VKORC1 enzyme converts the epoxide into reduced vitamin K; however warfarin inhibits this reaction. As a consequence, the physiologic role of vitamin K, which produces *γ*-carboxylation of several coagulation factors (prothrombin, factor VII, IX, and X), is inhibited.

Several groups have studied the role of VKORC1 in warfarin/acenocoumarol dose finding, dose maintenance, and bleeding risk associated with these drugs [33, 34, 59–61]. Only two studies have focused on patients receiving vitamin K antagonist following cardioembolic stroke. One found that the time and cumulative dosage of phenprocoumon needed to achieve target 2-3 INR ratio were associated with the presence of the *VKORC1 C283 + C837T* (*rs2359612*) polymorphism. Carriers of *TT* genotype needed shorter time to achieve target INR ratio (3.2 days) compared to *CC* carriers (6.5 days) [33]. The second paper evaluated the roles of *VKORC1*, gamma-glutamyl carboxylase (*GGCX*), calumenin (*CALU*), and cytochrome P450 2C9 (*CYP2C9*) in warfarin maintenance dose on Japanese stroke sufferers. Of the twelve SNPs analysed, the authors found that the *1639G > A, 3730G > A VKORC1* genotypes; the *8016G > A GGCX* genotype, and the *42613A > C CYP2C9* genotype were associated with dose maintenance. Thus, the variation in warfarin dose was explained for 33.3% by age, sex, weight, and three genetic polymorphisms (*VKORC1-1639G > A, CYP2C9 42613A > C, GGCX 8016G > A*). The importance of these loci has been recently confirmed using genome-wide association studies in acenocoumarol-treated patients [62, 63]. These studies found that the SNPs with the highest significance level were located in chromosome (cr.) 16 (rs10871454 and rs9923231) linked to VKORC1 and cr. 10 (rs4086116 and rs105791) linked with CYP2C9 gene. After adjusting for these two SNPs, two other polymorphisms reached significant association with acenocoumarol: rs2108622 within CYP4F2 gene on cr.19 and rs1995891 within CYP2C18 on cr. 10 [62, 63]. 

### 3.5. Antiplatelets

Antiplatelet drugs are commonly used treatment for ischemic noncardioembolic stroke [1].

#### 3.5.1. Aspirin

Aspirin is the more commonly used drug of this class, and its efficacy ranges between 13% and 25%. Its physiological role is to acetylate serine residue 530 in the active site of cyclooxygenase-1 (COX-1), sterically inhibiting the metabolism of arachidonic acid and consequently reducing thromboxane A2 (TxB2), which activates platelets. Numerous studies have investigated the genetic basis associated with recurrence of ischemic event in aspirin-treated patients (e.g., aspirin failure) (Table 4). 

COX-1 C50T allele has been correlated with a higher level of 11-dehydro-TxB2, both before and after aspirin; however, the haplotype studied did not confirm a genetic basis for aspirin failure [64, 65]. In addition, this polymorphism is not associated with a higher risk of stroke [39]. 

An interesting study compared the COX-gene sequence of patients with recurrent stroke (at least with two episodes) on aspirin and healthy subjects. The study found fourteen SNPs, and half of these lead to amino-acid substitutions; however, none of these variations was located near the COX catalytic site, thus this genetic polymorphisms could not explain the failure to respond to aspirin in this population of stroke patients [40].

#### 3.5.2. Clopidogrel

Clopidogrel is an oral, thienopyridine antiplatelet drug that irreversibly inhibits the P2Y12 subtype of ADP receptor, which has a major role in platelet aggregation. Clopidogrel has proven to be less effective in carriers of CYP2C19-reduced function allele [37, 38, 66]. These data have been confirmed in a recent meta-analysis that pooled 9 randomised trails for acute coronary syndrome or percutaneous coronary intervention; either homozygosis or heterozygosis carriers experience higher risk of stroke (Table 4) [35]. This could be caused by a relative reduction in the active metabolite of the drug, or by an insufficient inhibition of platelet aggregation. At a clinical level, CYP2C19 allele carriers have major adverse cardiovascular events, including stroke [35]. The TRITON TIMI 38 study on patients with acute coronary syndrome treated with PCI following clopidogrel versus another thienopyridine, “prasugrel,” explored the role of ABCB1, a glycoprotein that might affect clopidogrel transport and metabolism. The polymorphism on ABCB1 3435C → T was correlated to a significant increase in adverse outcome including cardiovascular death, MI, or stroke (*P* = 0.0064). Specifically, TT homozygote patients had a 72% increased risk of the primary endpoint compared with CT/CC individuals [36]. This result might be a consequence of the absolute reduction in maximum platelet aggregation that has been evidenced in healthy subjects enrolled in the same study [36]. Furthermore, the PLATO study explored the same polymorphisms in noncoronary artery bypass graft patients on clopidogrel versus ticagrelor, a novel ADP receptor blocker that does not need hepatic activation, and so is not influenced by CYP2C19 alleles. Patients on ticagrelor were less likely to experience stroke independently of CYP2C19 or ABCB1 genotype. In addition, no specific genotype-drug interaction was associated with any major bleeding risk [37]. Finally, an important GWA study has been performed on a healthy Amish population and found a positive association with clopidogrel response measured by ADP platelet aggregation percentage and 10q24 region (Table 4). This region contains CYP2C19∗2 genotype, which accounts for approximately 12% of the variation in clopidogrel response [38]. In addition, this study found a relevant association between this CYP2C19∗2 variant and event-free survival of adverse cardiovascular outcome in an independent population of 227 patients that underwent percutaneous coronary intervention [38]. 

## 4. Conclusions

Pharmacogenetics of stroke is a promising approach for optimizing treatment strategies aimed at decreasing stroke incidence and recurrence. Many candidate gene studies have examined the roles of polymorphisms on stroke treatment, and some of these have been replicated in GWA studies. However, few studies have considered stroke as an independent outcome, probably due to the relatively small number of events in the trails. 

Antihypertensive agents are the most extensively studied drug class. Some polymorphisms have been consistently identified but results remain controversial, probably due to differences in study designs and methods, small sample sizes, and short durations of follow-up [67].

Statins and stroke have failed to find any interaction with most of the studied polymorphism. In addition, GWAs that consider stroke as an outcome are not available. 

Tissue plasminogen activator has been investigated only in small studies on acute intravenous thrombolysis; thus, the pharmacogenetic data need to be reproduced in larger trails, and GWAs should be planned on humans in order to move forward in this field. Interestingly, a GWA study on ischemic rats has shown the genes regulated by rTPA treatment where different from the ones involved in ischemic stroke. In addition, gene expression profiles differed when reperfusion was or was not achieved [68]. If these results were to be replicated on humans, blood plasma could be used to monitor gene expression profiles, which are diversely associated with stroke and rtPA vessel recanalisation. 

Anticoagulants dose variability has been consistently reported to be explained by CYP2C9 and VKORC1 for the 33% and up to the 58% when adding clinical information [69]. For this reason, the United States Food and Drug Administration suggests testing for these two polymorphisms in order to achieve stable dose and to avoid major hemorrhagic events. Although the pharmacogenetic approach on warfarin is feasible in clinical practice [70]; its use in improving outcome (i.e., shorter time to achieve range INR, more stable dosing, greater percentage of time in therapeutic range, and lesser major bleeding events) over the classical clinical approach has been proven in only small samples [71, 72]; for this reason, larger studies (GIFT, COAG, and EU-PACT) are ongoing to demonstrate the usefulness of the genetic approach and clinical algorithms (see http://www.clinicaltrials.gov/) on outcome improvement. Unfortunately, genotype-guided warfarin dosing has not been demonstrated to be cost effective [73]. Finally, in the near future, vitamin K antagonists could be gradually replaced in many indications with the newer anticoagulants (e.g., Dabigatran, Apixaban, and Rivaroxaban), which do not require monitoring and dose adjustment [74–76].

Antiaggregants: genetic studies on aspirin failure in recurrent stroke patients have been unsuccessful in finding their genetic determinant. Several polymorphisms have been linked to poor clinical response to clopidogrel, but to date, no study has proven the usefulness of the pharmacogenetic approach with clopidogrel in improving outcome. For this reason, the ACCF/AHA [77] disagreed with the FDA regarding their decision to add a warning on clopidogrel label recommending genetic testing when prescribing it for the first time. However, several studies, focusing on coronary disease, not on stroke, are ongoing: GeCCO, RAPID GENE, TARGET PCI, and GIANT (see http://www.clinicaltrials.gov/).

The future availability and low cost of technology will allow for the screening of a large number of genetic determinants. This will lead to the description of polymorphisms that affect drug pharmacokinetics and dynamics in each given patient. Furthermore, this information will optimize the efficacy/toxicity ratio. Although promising, the results of pharmacogenetic studies need to be confirmed in prospective randomised trials of comparative effectiveness, comparing the classical clinical and the genotype-guided approach, before being used in clinical settings. Furthermore, no study has explored yet the clinical usefulness of the genetic approach in reducing adverse events. For these reasons, although promising, the genotype-guided approach for drug prescriptions is not routinely recommended [56].

## Figures and Tables

**Table 1:** Antihypertensive agents.

Name	Outcome	Gene and variant	Sample size/drugs used	Effect estimates and significance levels
INVEST-GENES [8]	Death/MI or stroke	GRK2 SNPs (rs1894111 *G* > *A*) GRK5 Gln41Leu	48/Verapamil SR, atenolol	GRK5 41Leu decreased the risk of adverse cardiovascular outcome adjusted independently of treatment (OR 0.535, 95% CI: 0.313–0.951)

INVEST-GENES [9]	Death/MI or stroke	KCNMB1 Glu65Lys KCNMB1 Val110Leu	5979 with HTN/Verapamil SR, atenolol	KCNMB1 110Leu had reduced risk of composite outcome (HR 0.68 (95% CI 0.47–0.998); this effect was higher in Verapamil SR (HR 0.587, 95% CI 0.33–1.04) than atenolol-treated patients (HR 0.946, 95% CI 0.56–1.59)

LIFE substudy [10]	Cardiovascular events	13 polymorphisms (angiotensin-converting enzyme I/D, *α*-adducin Gly460Trp, *β*1-adrenergic receptor Gly389Arg, *β*2-adrenergic receptor Arg16Gly, *β*2-adrenergic receptor Gln27Glu, *β*3-adrenergic receptor Trp64Arg, angiotensinogen Met235Thr, aldosterone synthase promoter C-344T, type 1 angiotensinogen receptor A1166C, bradykinin 2 receptor I/D, CYP2C9∗2 versus CYP2C9∗1, CYP2C9∗3 versus CYP2C9∗1, G protein *β*3-subunit C825T)	3503/Losartan, atenolol	No significant genotype-drug interaction on the outcome

GEN-HAT [11]	Primary: fatal CHD/nonfatal MI. Secondary: stroke, all-cause mortality, combined CHD, and combined cardiovascular disease	ACE I/D	37,939/chlorthalidone, amlodipine, lisinopril, or doxazosin	No significant association with the outcome was reported; no significant gene-drug interaction was found

GEN-HAT [12]	Primary: fatal CHD/nonfatal MI. Secondary: stroke, all-cause mortality, combined CHD, and 6-mos systolic and diastolic BP changes	NPPA SNP T2238C (rs5065) NPPA SNP G664A (rs5063)	38,462 with HTN/chlorthalidone, amlodipine, lisinopril, or doxazosin	NPPA T2238C TT variant *x* “chlorthalidone versus amlodipine” interaction was significantly associated stroke (HR 1.09 95% CI 0.95–1.26). NPPA T2238C TT variant *x* “chlorthalidone versus amlodipine + lisinopril” interaction was significantly associated with stroke (HR 1.09 95% CI 0.95–1.26). NPPA T2238C CC variant *x* “chlorthalidone versus amlodipine” interaction was significantly associated with stroke (HR 1.18 95% CI 0.72–1.90). Either NPPA T2238C variant or NPPA G664A was not significantly associated with stroke and “chlorthalidone versus lisinopril,” “chlorthalidone versus doxazosin”

GEN-HAT [13]	Primary: fatal CHD/nonfatal MI. Secondary: stroke, heart failure, all-cause mortality, end-stage renal disease	FGB G455A	30 076 with HTN/chlorthalidone, amlodipine, lisinopril	Common GG homozygotes had higher stroke risk on lisinopril versus amlodipine (HR 1.38, *P* < 0.001); variant A allele carriers had slightly lower risk on lisinopril versus amlodipine (HR 0.96, *P* value for interaction = 0.03)

Lemaitre et al. [14]	MI, ischemic stroke	ADRB1 (Seven SNPs plus haplotype), ADRB2 (five SNPs plus haplotypes)	938 cases with MI or stroke/beta blocker	beta1-AR gene variation and beta-blocker use showed a positive interaction on ischemic stroke risk (*P* = 0.04). Homozygosis or heterozygosis for rs#2429511 variant was associated with higher MI/stroke combined risk in beta-blocker users (OR 1.24 95% CI 1.03–1.50). No interaction of ADRB2 with beta-blocker use and outcomes

Rotterdam study [15]	MI, stroke	AGT (M235T)	4097 with HTN/ACEI, BB	No significant gene-drug interaction was found on stroke

Rotterdam study [16]	MI, stroke	AGTR1 (C573T) ACE (I/D)	4097 with HTN/ACEI, BB	No significant AGTR1 and ACE I/D interaction on stroke risk with ACEI or BB

INVEST-GENES [17]	Death/nonfatal MI/nonfatal stroke	ADRB1 (Ser49GLy, Arg389Gly) and ADRB2 (Gly16Arg, Gln27Glu, 523 C > A)	5,895 CAD patients/Verapamil SR, atenolol	No association between any haplotype and treatment on stroke

INVEST-GENES [18]	Death/nonfatal MI/nonfatal stroke	NOS3-786T > C (rs2070744), NOS3 Glu298 > Asp (rs1799983)	258 death/MI/stroke versus 774 control	No genetic interaction with drugs and composite outcome

Psaty et al. [19]	MI/nonfatal stroke	ADD1 (Gly460Trp)	Cases versus controls	ADD1 Trp460 variant had lower stroke risk on diuretics (OR, 0.49; 95% CI, 0.32–0.77). The point estimate of diuretic-adducin interaction was SI 0.45 (95% CI 0.26–0.79) for the combined outcome MI and stroke; separate analyses yielded similar results: MI (SI 0.41 95% CI 0.21–0.80) and stroke (SI 0.53 95% CI 0.24–1.19)

INVEST-GENES [20]	Death/nonfatal MI/nonfatal stroke	ADD1 Gly460Trp	5,979 CAD patients/Verapamil SR, atenolol	ADD1 Trp460 black carriers had higher combined outcome risk (aHR 2.62, 95% CI 1.23–5.58), compared to whites (aHR 1.24 95% CI 0.90–1.71) and Hispanics (aHR 1.43 95% CI 0.86–2.39). No significant interaction between the ADD1 polymorphism and diuretic use for either primary outcome or secondary outcomes

PROGRESS [21]	Fatal and nonfatal stroke (ischemic or hemorrhagic), nonfatal MI/coronary death, composite nonfatal stroke/nonfatal MI/vascular death, all-cause mortality, dementia, and cognitive decline	ACE I/D	5688 with stroke or TIA/perindopril	No associations between ACE genotypes and cerebrovascular disease history or cardiovascular risk factors was demonstrated. The ACE genotype was not associated with the long-term risks of stroke. The ACE genotype did not modify the relative benefits of perindopril over placebo

**Table 2:** Statins.

Author's name/study name	Outcome	Gene (variant)	Sample size/drug	Effect estimates and findings
Hindorff et al. [22]	Nonfatal MI/nonfatal stroke	ABCB1, CETP, HMGCR, LDLR, LIPC, NOS3	865 with MI, 368 with stroke and 2686 controls/statins	No gene-statin interactions for stroke. 5 SNP-statin interactions on stroke (one CETP, four LIPC); no gene level association for stroke; SNP level association: two SNPs (one CETP, one LDLR) for stroke. The highest significance was found for stroke in CETP rs5883 carriers on simvastatin (OR 3.60 95% CI 1.22–7.70)

Heart protection study [23]	Major coronary event (coronary death or nonfatal MI), major vascular event (major coronary event plus revascularization or stroke)	KIF6 Trp719Arg polymorphism (rs20455) on vascular risk and response to statin therapy in from of the heart protection study	18,348 participants/simvastatin	No significant gene-statin interaction with any of the outcome, including stroke

Rotterdam study [24]	Death, MI, stroke	Apo E (E2, E3, E4)	7983 older than 55 yo/statins	No significant gene-statin interaction with any of the outcome. Statins reduce stroke risk (aOR 0.50 95% CI 0.28–0.91) independently of Apo E genotype

GenHAT [25]	Primary outcome: all-cause mortality, secondary outcomes (fatal CHD and nonfatal MI, CVD mortality, CHD, stroke, other CVD, non-CVD mortality, stroke, and heart failure)	ACE (I/D)	9467/pravastatin	No significant gene-statin interaction with any of the outcome

**Table 3:** Tissue plasminogen activator.

Author's name/study name	Outcome	Gene (Variant)	Sample Size/drug	Effect estimates and findings
Broderick et al. 2001 [26]	Favourable outcome (NIHSS of 0 or 1, Barthel Index of 95 or 100, Modified Rankin Scale of 0 or 1, and a Glasgow Outcome Scale of 1.)	ApoE (E2, E3, E4)	409 ischemic stroke/rTPA versus PB within 3 hours	ApoE E2 phenotype-rt-PA interaction was associated with good outcome at 3 months (OR: 6.4; 95% CI: 2.7–15.3). Apo E4 phenotype not related to favorable 3 month outcome, response to t-PA, 3-month mortality, or risk of intracerebral hemorrhage

Fernández-Cadenas et al. [27]	Recanalization rate, NIHSS at 48 hours and mRS score at 3 months, heamorrhagic transformation	ApoE (E2, E3, E4)	77 ischemic stroke/rTPA within 3 hours	No significant association of ApoE genotype and the studied outcome

Del Río Espínola et al. [28]	Reocclusion rate	236 SNPs form candidate genes for vascular risk factor	222 ischemic stroke/rTPA	rs1883832 SNP from CD40 gene (OR: 0.077; 95% CI: 0.009–0.66) and rs1800801 SNP from the MGP gene (OR 15.25; 95% CI: 2.23–104.46) were independently associated with reocclusion after adjustment for clinical predictors

Fernández-Cadenas et al. [29]	Recanalization	ACE (I/D)	96 ischemic stroke/rTPA within 3 hours	ACE DD homozygosis was significantly associated with recanalization rate following rTPA (OR: 4.3 95% CI: 1.35–13.49). No relation was found between ACE I/D polymorphism and symptomatic hemorrhagic complications. No association between ACE genotypes and Factor VII or Factor X activities

Fernández-Cadenas et al. [30]	Recanalization	PAI-1 4G/5G TAFI (Thr325Ile)	139 with ischemic stroke/TPA within 3 hours	PAI-1 4 *G*/5 *G* was not associated with recanalization. TAFI Thr325Ile polymorphism was associated with recanalization resistance (OR 5.6 95% CI 1.2–20). Combination of TAFI and PAI-1 polymorphisms double the risk of absence of recanalization (OR: 11.1; 95% CI: 1.4–89.8)

González-Conejero et al. [31]	Death, recanalization	Factor XIII (FXIII) V34L	200 with ischemic stroke/TPA within 3 hours	FXIII 34 L carriers had higher death risk than V/V (OR 2.50 95% CI 1.00–7.06); high fibrinogen levels higher risk than lower levels (OR 2.72 95% CI 1.01–7.44); FXIII 34L and high fibrinogen level higher risk than FXII V and low fibrinogen (OR 5.74 95% CI 1.51–11.56). No difference in recanalization rate

Montaner et al. [32]	Hemorrhagic transformation	MMP9 (C1562T)	61 with ischemic stroke/TPA within 3 hours	The polymorphism studied does not increase hemorrhagic risk

**Table 4:** Anticoagulants and antiplatelets.

Author's name/study name	Outcome	Gene (Variant)	Sample size/drug	Effect estimates and findings
*Anticoagulants*				
Arnold et al. [33]	Dose finding	VKORC1 C283 + 837C → T (rs2359612)	49 with cerebrovascular disease/phenprocoumon	VKORC1 TT carriers reached an INR of 2-3 after a mean time of 3.2 days (*n* = 5), CT carriers after 4.4 days (*n* = 27), and CC carriers after 6.5 days (*n* = 15)
Kimura et al. [34]	Warfarin maintenance dose	(VKORC1), gamma-glutamyl carboxylase (GGCX), calumenin (CALU), and cytochrome P450 2C9 (CYP2C9)	93 Japanese on stable anticoagulation therapy	1639 *G* > *A* (*P* = 0.004) and 3730 *G* > *A* genotypes (*P* = 0.006) in VKORC1, the 8016 *G* > *A* genotype in GGCX (*P* = 0.022), and the 42613 *A* > *C* genotype in CYP2C9 (*P* = 0.015) were associated with effective warfarin dose
*Antiplatelets*				
Meta-analysis of 9 different studies (CLARITY TIMI 28, EXCLESIOR, TRITION TIMI 38, AFIJI, FASSTS-MI, RECLOSE, ISAR, CLEAR PLATELETS, Intermountain) [35]	Composite outcome (cardiovascular death/MI/stroke) and stent thrombosis	CYP2C19/1 or 2 reduced function alleles (∗2, ∗3, ∗4, ∗5, ∗6, ∗7, and ∗8)	9685 patients (91% had PCI, 54% had ACS)/clopidogrel	Carriers of 1 (HR 1.55; 95% CI, 1.11–2.17) or 2 (HR 1.76; 95% CI, 1.24–2.50) reduced-function CYP2C19 alleles had higher risk of composite outcome events
TRITON-TIMI 38 [36]	Composite outcome (cardiovascular death/MI/ischemic stroke)	CYP2C19/1 or 2 reduced function alleles (∗2, ∗3, ∗4, ∗5, ∗6, ∗7, and ∗8) ABCB1/3435C → T	2932 patients with ACS undergoing PCI/clopidogrel versus prasugrel	TT homozygotes of ABCB1 genotype had increased risk of the composite outcome compared to CT or CC carriers (HR 1.72, 95% CI 1.22–2.44). Carriers of a CYP2C19 reduced-function allele only (Kaplan-Meier event rate 11.5%), ABCB1 3435 TT homozygotes only (Kaplan-Meier event rate 12.6%), or both (Kaplan-Meier event rate 13.6%) had increased risk of composite outcome (pooled HR 1.97, 95% CI 1.38–2.82). No significant genotype-prasugrel interaction was reported
PLATO [37]	Composite outcome (cardiovascular death/MI/stroke)	CYP2C19/1 or 2 reduced function alleles ABCB1/3435C → T	10285 patients with ACS undergoing non-CABG/clopidogrel versus ticagrelor	Either with (HR 0.77, 95% CI 0.60–0.99) and without (0.86, 0.74–1.01, *P* = 0.0608) any CYP2C19 reduced-function alleles patients on ticagrelor experienced lower risk of composite outcome compared to patients on clopidogrel (interaction *P* = 0.46). Independently of ABCB1 genotype, patients on ticagrelor had lower risk of the composite outcome compared to clopidogrel users (interaction *P* = 0.39; HR 0.71, 95% CI 0.55–0.92). No significant interaction was found on treatment and genotype regarding major bleeding
PAPI study and Mount Sinai study [38]	Composite outcome (cardiovascular death, MI, ischemic stroke, stent thrombosis, unplanned target vessel revascularization, unplanned nontarget vessel revascularization, hospitalization for coronary ischemia)	GWA	429 white healthy Amish individuals/clopidogrel; results replicated in 227 undergoing PCI	13 SNPs in 10q24 region, where CYP2C18–CYP2C19–CYP2C9–CYP2C8 gene cluster is found, were associated with reduced response to clopidogrel. CYP2C19∗2 allele carriers were at higher risk for composite outcome (adjusted HR 2.42 95% CI 1.18–4.99)
Clappers et al. [39]	Composite outcome (cardiovascular death/MI/stroke)	COX-1/C50T	496 admitted to Coronary Care Unit for different reasons/aspirin	No interaction was found on genotype and aspirin for the composite outcome
Hillarp et al. [40]	n.a.	COX-1/C116T, del 137–142, C144T, G6841A, G7331C, A7742A, C10427A, C10608A, del 10675A, G12254A, T12378C, G19187A, C19242T, G19255A	68 with recurrent stroke/ASA	14 variants of the Cox-1 gene were identified and 7 involved amino acid substitutions of the Cox-1 molecule. None of the mutations were located near the catalytic site

ABCB1: ATP-binding cassette subfamily B, ACEI: angiotensin convertin enzyme inhibitors, ACE I/D: angiotensin convertin enzyme insertion/deletion, ACS: acute coronary syndrome, ADD1: *α*-adducin, ADRB: *β*-adrenergic receptor, AGT: angiotensinogen, AGTR1: angiotensin receptor II type 1, APO E: apolipoprotein E, BP: blood pressure, CABG: coronary artery bypass graft, CAD: coronary heart disease, CD: cluster of differentiation, CEPT: cholesteryl ester transfer protein, CHD: coronary artery disease, COX: cyclooxygenase, CVD: cerebrovascular disease, CYP: cytochrome P, FGB: fibrinogen beta, GRK: G-protein-coupled receptor kinase, GWA: genome-wide association, HMG-CoR: hydroxyl-methylcoenzyme A reductase, HR: hazard ratio, HTN: hypertension, KCNMB: conductance calcium and voltage-dependent potassium channel, KIN 6: kinesin family member 6, LDLR: low-density lipoprotein receptor, LIPC: human hepatic lipase, MGP: matrix Gla protein, MI: myocardial infarction, MMP: matrix metalloproteinase, NIHSS: National Institute of Health stroke scale, NOS: nitric oxide synthase, NPPA: atrial natriuetic polypeptide precursor, OR: odds ratio, PAI: plasminogen activator inhibitor, PCI: percutaneous coronary intervention, SI: sinergy index, TAFI: thrombin-activable fibrinolysis inhibitor, verapamil SR: verapamil-sustained release, VKORC1: vitamin K epoxide reductase complex subunit 1.
